# Supplementary material for: Sequential Extraction of Proanthocyanidin Fractions from Ficus Species and Their Effects on Rumen Enzyme Activities In Vitro
Source: Molecules. 2022 Aug 12;27(16):5153. doi: 10.3390/molecules27165153 (PMC9415173; doi:10.3390/molecules27165153)
Supplement: Supplementary file 1 [file molecules-27-05153-s001.zip › molecules-1831411-supplementary.pdf]

Supplementary figures and tables

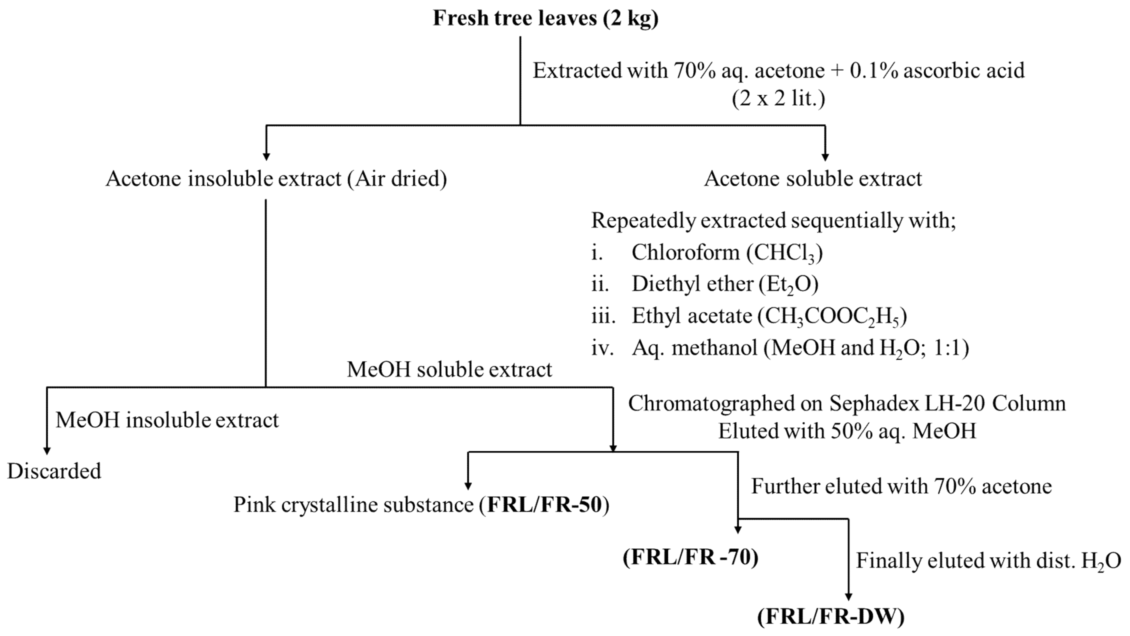

Figure S1. Schematic flowchart for extraction and isolation of proanthocyanidins from tree leaves

Table S1. Stereochemistry of flavan-3-ols in with hydroxylation pattern and % of extender unit and terminal units in oligomers isolated from tree leaves

Fractions	(+) Catechin		(-) Epicatechin		(+) Gallocatechin		(-) Epigallocatechin		Extender unit (%)	Terminal unit (%)
	Position of ‘OH’	*R-S* configuration	Position of ‘OH’	*R-S* configuration	Position of ‘OH’	*R-S* configuration	Position of ‘OH’	*R-S* configuration		
FRL-50	3,5,7,3'4'	2*R*: 3*S*	3,5,7,3'4'	2*R*: 3*R*	3,5,7,3'4'5'	2*R*: 3*S*	3,5,7,3'4'5'	2*R*: 3*R*	76	24
FRL-DW	-	-	3,5,7,3'4'	2*R*: 3*R*	3,5,7,3'4'5'	2*R*: 3*S*	3,5,7,3'4'5'	2*R*: 3*R*	80	20
FR-70	3,5,7,3'4'	2*R*: 3*S*	3,5,7,3'4'	2*R*: 3*R*	3,5,7,3'4'5'	2*R*: 3*S*	3,5,7,3'4'5'	2*R*: 3*R*	84	16
FR-50	-	-	3,5,7,3'4'	2*R*: 3*R*	3,5,7,3'4'5'	2*R*: 3*S*	3,5,7,3'4'5'	2*R*: 3*R*	78	22
FR-DW	3,5,7,3'4'	2*R*: 3*S*	3,5,7,3'4'	2*R*: 3*R*	3,5,7,3'4'5'	2*R*: 3*S*	3,5,7,3'4'5'	2*R*: 3*R*	68	32

FRL-50: Extract eluted at 50% of methanol from *Ficus religiosa,* FRL-DW: Extract eluted with distilled water from *F. religiosa*, FR-70: Extract eluted at 70% of acetone from *F. racemosa*, FR-50: Extract eluted at 50% of methanol from *F. racemosa,* FR-DW: Extract eluted with distilled water from *F. racemosa.*

R-S configuration: It is used to differentiate between two enantiomers of a chiral molecule.
